# Amyloid‐β PET in Alzheimer's disease: A systematic review and Bayesian meta‐analysis

**DOI:** 10.1002/brb3.2850

**Published:** 2022-12-27

**Authors:** Dan Ruan, Long Sun

**Affiliations:** ^1^ Department of Nuclear Medicine Zhongshan Hospital (Xiamen), Fudan University Fujian China; ^2^ Department of Nuclear Medicine and Minnan PET Center Xiamen Cancer Hospital, The First Affiliated Hospital of Xiamen University Xiamen China

**Keywords:** ^11^C‐PIB, ^18^F‐florbetapir, Alzheimer's disease, amyloid‐β PET, MCI converting to AD

## Abstract

**Background:**

In recent years, longitudinal studies of Alzheimer's disease (AD) have been successively concluded. Our aim is to determine the efficacy of amyloid‐β (Aβ) PET in diagnosing AD and early prediction of mild cognitive impairment (MCI) converting to AD. By pooling studies from different centers to explore in‐depth whether diagnostic performance varies by population type, radiotracer type, and diagnostic approach, thus providing a more comprehensive theoretical basis for the subsequent widespread application of Aβ PET in the clinical setting.

**Methods:**

Relevant studies were searched through PubMed. The pooled sensitivities, specificities, DOR, and the summary ROC curve were obtained based on a Bayesian random‐effects model.

**Results:**

Forty‐eight studies, including 5967 patients, were included. Overall, the pooled sensitivity, specificity, DOR, and AUC of Aβ PET for diagnosing AD were 0.90, 0.80, 35.68, and 0.91, respectively. Subgroup analysis showed that Aβ PET had high sensitivity (0.91) and specificity (0.81) for differentiating AD from normal controls but very poor specificity (0.49) for determining AD from MCI. The pooled sensitivity and specificity were 0.84 and 0.62, respectively, for predicting the conversion of MCI to AD. The differences in diagnostic efficacy between visual assessment and quantitative analysis and between ^11^C‐PIB PET and ^18^F‐florbetapir PET were insignificant.

**Conclusions:**

The overall performance of Aβ PET in diagnosing AD is favorable, but the differentiation between MCI and AD patients should consider that some MCI may be at risk of conversion to AD and may be misdiagnosed. A multimodal diagnostic approach and machine learning analysis may be effective in improving diagnostic accuracy.

## Graphical abstract

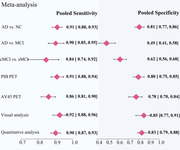

## 1 INTRODUCTION

Alzheimer's disease (AD) is the most common type of dementia. The prevailing AD pathogenesis hypothesis suggests that AD is mainly due to the accumulation of insoluble amyloid‐β (Aβ) deposits and neurofibrillary tangles induced by highly phosphorylated tau (p‐τ) proteins in the neocortex, hippocampus, and amygdala, accompanied by massive loss of neurons and synapses leading to brain atrophy (Duyckaerts et al., 2009; Markesbery, 1997; Mirra et al., 1991). Recent epidemiological surveys of AD patients in the United States show that about 6.2 million people over the age of 65 will suffer from AD in 2021. Frighteningly, the number of people with AD is expected to increase significantly to 13.8 million by 2060 as the population ages and if there are no effective prevention and treatment methods (Alzheimer's Association, 2021). AD is now regarded as a chronic disease, which brings great emotional and economic burdens to individuals, families, and society, and is receiving increasing attention from the medical community. Early and accurate diagnosis, effective prevention, and treatment of AD are the most sought‐after challenges to overcome.

Cognitive decline and the deposition of Aβ plaques in AD are progressive and evolving, whereas Aβ deposition mostly occurs before symptoms; therefore, early AD may present as a preclinical stage of normal cognition or mild cognitive impairment (MCI). MCI is a state between normal cognition and dementia. Not all MCI progresses to AD or dementia, and even after 10 years, a significant proportion of these patients remain stable or even return to normal cognition during follow‐up. It has been shown that approximately 6%–16.5% of MCI patients convert to AD each year (Bruscoli & Lovestone, 2004; Ward et al., 2013). Identifying patients with MCI who will develop AD is key to preventing its onset and progression and treating it early.

The gold standard for diagnosing AD is brain tissue pathology, usually an autopsy of the brain performed after the patient's death. However, performing brain biopsies on living patients to determine if they have AD is not ethically supported and can lead to brain tissue damage and more severe cognitive impairment. Brain biopsy is often replaced by mental status examinations and neuropsychological testing batteries in clinical practice. NINCDS‐ADRDA and DSM‐IV are commonly used clinical diagnostic criteria, with an approximate accuracy range of 65%–96% (Dubois et al., 2007; Mckhann et al.,^1984^; Alzheimer's Association, 2021). Results from some AD autopsies have shown that the clinical diagnosis of AD has a positive predictive value of 0.91 for eventual pathologically definitive AD (Chui & Lee, 2003). More than 30 years ago, it was proposed that cognitive decline in AD is associated with histopathological changes in the hippocampus, of which hippocampal atrophy is often considered an early feature of the degeneration of consciousness in AD patients (Ball et al., 1985). Visual assessment of hippocampal atrophy has approximately 80%–85% sensitivity and specificity for AD diagnosis. In addition, several studies have predicted the progression of MCI to AD by measuring hippocampal volume on MRI images or by assessing the degree of brain atrophy, but all have shown poor results (Frisoni et al., 2010; Yuan et al., 2009). ^18^F‐FDG PET has been used for more than two decades as an aid in diagnosing AD, characterized by hypo‐glucose metabolism in the temporoparietal and posterior cingulate regions (Herholz et al., 2002). Decreased glucose metabolism in the brain occurs before macroscopic brain atrophy is observed; thus, ^18^F‐FDG PET is thought to have the potential to detect early neurodegenerative changes earlier and more sensitively than MRI (Kljajevic et al., 2014). Multicenter studies have shown that ^18^F‐FDG PET can correctly classify 95% of AD patients but has average efficacy in predicting the conversion of MCI to AD (Shaffer et al., 2013). Regardless, the brain metabolic rate revealed by ^18^F‐FDG PET emphasizes the degree of neuronal activity and does not elucidate the underlying pathogenic neuropathological changes.

In contrast, the radiotracers indicating Aβ loading, p‐τ protein aggregation, and neuroinflammation are more likely to directly respond to the pathological status of patients with AD and MCI converting to AD (cMCI) (Chandra et al., 2019). Aβ‐specific PET has been used clinically, whereas radionuclides specific to p‐τ protein and neuroinflammation have not been recommended for clinical application. A strong correlation between ^11^C‐PIB retention in the brain and pathology (neuroinflammatory plaques and vascular amyloid) at autopsy has been reported early on (Ikonomovic et al., 2008). In recent years, the most commonly used Aβ tracer is ^11^C‐PIB, which can differentiate between mild AD and healthy controls (Chandra et al., 2019). By determining the retention of ^11^C‐PIB in the frontal, temporal, and cingulate cortices, predicting the conversion of MCI to AD can also be easily done (Brück et al., 2013). On the other hand, the degree of gray matter atrophy, regional brain glucose metabolic rate, and Aβ deposition differed between MCI converting to AD (cMCI) and stable MCI (sMCI). During the gradual progression of MCI to AD, Aβ deposition precedes gray matter atrophy and decreased brain glucose metabolism (Jack et al., 2013; Ly et al., 2010). The utilization of ^11^C‐PIB in cyclotron‐free clinical centers is limited by its short half‐life, including the difficulty of transport, storage, and substantial waste of resources. Therefore, novel ^18^F‐labeled amyloid tracers ^18^F‐florbetapir, ^18^F‐florbetaben, and ^18^F‐flutemetamol (^18^F‐FMM) could overcome the abovementioned nontechnical limitations. ^18^F‐FMM is a derivative of ^11^C‐PIB, which has similar biological properties to ^11^C‐PIB and has essentially the same uptake in the cerebral cortex (Nelissen et al., 2009; Vandenberghe et al., 2010). ^18^F‐florbetapir (^18^F‐AV45) was approved by the FDA in 2011 as a radiotracer to aid in diagnosing AD (Yang et al., 2012). Based on a preliminary literature search, most research on brain Aβ PET is concentrated in developed countries, whereas clinical centers in developing countries have applied Aβ PET less frequently or are still in experimental research phase. Our study comprehensively assesses the diagnostic performance of Aβ PET by pooling a considerable number of reliable studies and explores the overall trends in the performance of Aβ PET in AD over the past decade.

## 2 MATERIALS AND METHODS

We conducted this systematic review and meta‐analysis in strict compliance with the Preferred Reporting Items for Systematic Reviews and Meta‐analysis (PRISMA) 2020 statement (Page et al., 2021). The steps for an inclusion of the literature were performed according to the 2020 PRISMA flow diagram. The methodological quality evaluation of our included literature was performed by referring to the Cochrane Handbook for Systematic Reviews of Diagnostic Test Accuracy and the entries of Quality Assessment of Diagnostic Accuracy Studies‐2 (QUADAS‐2) (Deeks et al., 2021; Whiting, 2011).

### 2.1 Search strategy and study selection

We searched all the literature on Aβ PET for AD diagnosis in the PubMed database with a deadline of January 1, 2022. The main terms we used for the search were as follows: Amyloid PET or ^11^C‐PIB or ^18^F‐AV45 PET and AD. Our inclusion criteria for the study were as follows: Aβ PET for diagnosing AD or MCI progression to AD, PET as a tool for diagnosis. Exclusion criteria were as follows: reviews, case reports, commentaries, and editorials; the number of cases less than 5; inability to extract diagnostic data; inaccurate diagnostic data extracted from the study; and presence of other systemic or brain diseases. We excluded a portion of the literature by gradually adding conditional filters based on the exclusion criteria. Then, we further excluded a portion of the literature by reading the title, abstract, and full text.

### 2.2 Data selection and quality assessment

The information we extracted for the literature was finally included in our meta‐analysis, including general information about the studies, patient characteristics, main study objectives, type of radiotracer, diagnostic approach, and reference standard. In addition, we extracted information about patient selection, diagnostic approach, and reference standard in each study according to the evaluation entries of QUADAS‐2. For the assessment of patient selection, if case control, we considered that these studies introduced a high risk of bias. For unblinded studies or studies that did not mention whether they were blinded to clinical information, we combined the diagnostic approach and the way the threshold was set to determine whether there was a risk of bias in the index test. In assessing the reference standard, we classified all unblinded studies as unclear risks. For some articles, the primary study objective of which was not to diagnose AD with PET, we evaluated them as unclear risk in the patient selection and study flow.

The two authors worked together to extract diagnostic data and study‐related characteristic information and independently assess the methodological quality of the studies, and any disagreements were resolved through discussion.

### 2.3 Statistical analysis

There may be substantial heterogeneity between studies as we included studies with different diagnostic methods and diagnostic thresholds. We performed a meta‐analysis using the Bayesian bivariate analysis based on the integrated nested Laplace approximation method, which fully considered the heterogeneity among studies and the interrelatedness and interplay of sensitivity and specificity. The analysis software we used was R (R for Windows, version 4.1.0). We calculated the pooled sensitivity, specificity, DOR, and 95% confidence interval, respectively, and fitted the summary ROC curve based on the binomial–normal model. Finally, a funnel plot was plotted to assess publication bias and heterogeneity.

## 3 RESULTS

### 3.1 Literature search and study characteristics

**FIGURE 1:**
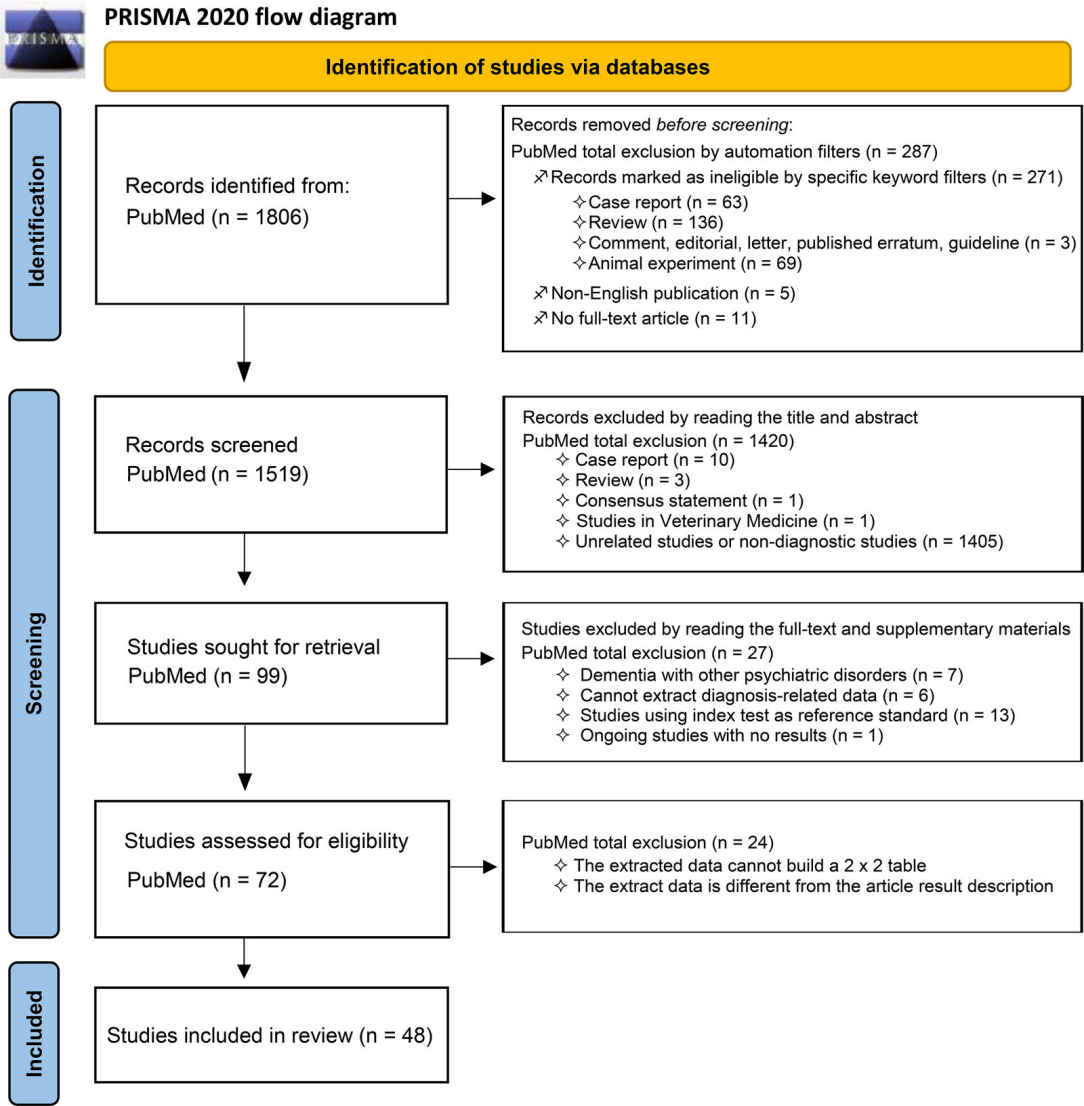
Flowchart of the study screening process following the Preferred Reporting Items for Systematic Reviews and Meta‐analysis (PRISMA) 2020 statement

First, 1806 publications were retrieved without adding any conditional filters. Two hundred eighty‐seven articles were excluded through the filter at the official PubMed website, and 1420 articles were further excluded by reading the titles and abstracts. After carefully reading the full text and supplementary materials and judging the accuracy and reliability of the extracted data, the final number of articles included in our study was 48 (Beach et al., 2014; Brück et al., 2013; Camus et al., 2012; Clark et al., 2012; Fleisher, 2011; Hatashita & Yamasaki, 2013; Hatashita et al., 2014; Hosokawa et al., 2015; Jack et al., 2012; Kaneko et al., 2014; Kerbage et al., 2015; Li et al., 2015; Mattsson et al., 2014; Mikhno et al., 2012; Newberg et al., 2012; Ng et al., 2007; Rabinovici et al., 2011; Saint‐Aubert et al., 2014; Tolboom et al., 2010; Trzepacz et al., 2014; Tzen et al., 2014; Vandenberghe et al., 2010; Villemagne et al., 2011, 2019; Alvarez et al., 2018; Ben Bouallegue et al., 2017; Chen et al., 2016; Dukart et al., 2016; Iaccarino et al., 2017; La Joie et al., 2019; Mielke et al., 2018; Oliveira et al., 2018; Ottoy et al., 2019; Park et al., 2019; Schreiber et al., 2015; Seo et al., 2017; Takahashi et al., 2017; Villeneuve et al., 2015; Wang, Chen, et al., 2016; Wang, Yi, et al., 2016; Xu et al., 2016; Zhang et al., 2017; Zwan et al., 2016, 2021; Chanisa et al., 2021; Kitajima et al., 2021; Lesman‐Segev et al., 2021; Peretti et al., 2019). The specific process of literature screening is detailed in Figure 1.

**FIGURE 2:**
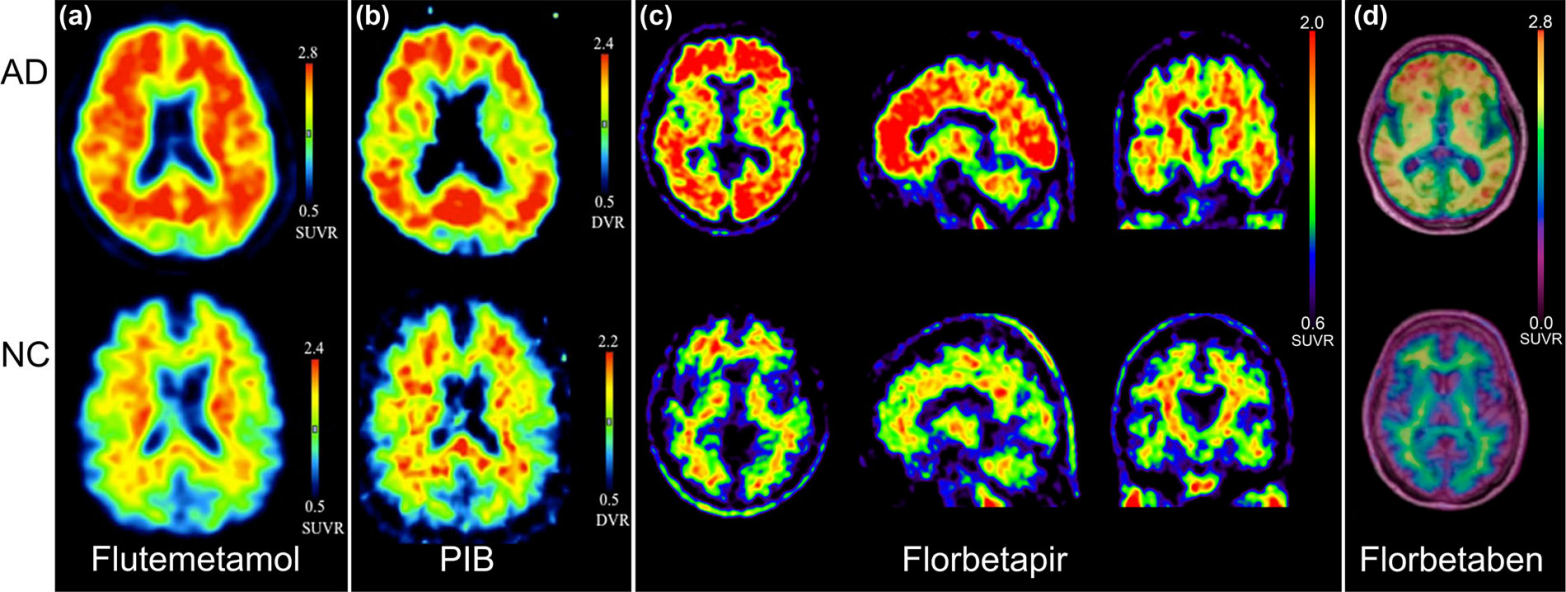
The typical brain images of Alzheimer's disease (AD) and normal controls are shown by amyloid‐β (Aβ) PET imaging. Part (a) shows axial ^18^F‐flutemetamol (^18^F‐FMM) PET images (upper row for AD patients, lower row for normal controls). Part (b) shows axial ^11^C‐PIB PET images (upper row for AD patients, lower row for normal controls). Part (c) shows ^18^F‐AV45 PET images in transaxial, sagittal, and coronal positions (upper row for AD patients, lower row for normal controls). Part (d) shows axial ^18^F‐florbetaben PET images with aligned fused MRI images (upper row for AD patients, lower row for normal controls). *Source*: From Camus et al. (2012, Hatashita et al. (2014)), and Villemagne et al. (2011) with modifications

A total of 8285 patients were included in the 48 studies; the number of patients included in the analysis in our meta‐analysis was 5967. The types of populations included in the studies had AD, MCI, non‐AD dementia (non‐ADD), and normal controls (NC), and their median age range was 60.1–79.4 years. The radiotracers were ^11^C‐PIB or ^18^F‐AV45, except for one study that used ^18^F‐florbetaben and three studies that used ^18^F‐FMM. The typical comparison of the uptake of ^18^F‐FMM, ^11^C‐PIB, ^18^F‐AV45, and ^18^F‐florbetaben in AD patients and NC is shown in Figure 2. Six studies had a reference standard of brain autopsy or brain tissue biopsy, and the remaining 42 studies had a reference standard of comprehensive clinical diagnostic criteria. One study included only patients with AD; the remaining studies compared different population groups, including AD versus NC, AD versus MCI, cMCI (MCI converting to AD) versus sMCI (stable MCI), AD versus FLTD, and AD versus non‐ADD. Detailed information on these included studies is shown in Table 1.

**TABLE 1:** Basic characteristic information for the included studies

Year	Author	Ref	Patients’ origin	Study design	Major research objectives	*N* of pts	Types of patients	Age (median/range) in years	Male/female	Test index	Qualitative/visual analysis	Threshold	Reference standard	Interval time (PET to autopsy/follow‐up) mean/range	Extracted diagnostic objects
2007	Ng S	Ng et al. (2007)	Australia	Prosp	Diagnostic value of PIB and FDG for AD	40	15 AD, 25 NC	71.6	21/19	PIB	V&Q	SUVR = 1.54	NINCDS‐ADRDA, MMSE, CDR	NA	AD vs. NC
2010	Tolboom N	Tolboom et al. (2010)	Netherlands	Prosp	Diagnostic value of PIB for AD	41	21 AD, 20 NC	65	NR	PIB	V&Q	^b^BPnd = 0.54	NR	NA	AD vs. NC
2010	Vandenberghe R	Vandenberghe et al. (2010)	Belgium	Prosp	Diagnostic value of FMM for AD	72	27 AD, 20 MCI, 25 NC	65.9	36/36	FMM	V&Q	SUVR = 1.56	NINCDS‐ADRDA, ADAS‐cog, MMSE, CDR, DSM‐IV, FCSRT, RAVLT	1 month to 6 years	AD vs. NC; AD vs. MCI
2011	Fleisher AS	Fleisher (2011)	USA	Prosp	Diagnostic value of AV45 for AD	210	68 AD, 60 MC, 82 NC	72.7	99/111	AV45	Q	SUVR = 1.17	NINCDS‐ADRDA, ADAS‐cog, MMSE, CDR, WMS‐imd	NA	AD vs. NC; AD vs. MCI
2011	Rabinovici GD	Rabinovici et al. (2011)	USA	Prosp	Comparing the diagnostic efficacy of PIB and FDG for AD and FTLD	107	62 AD, 45 FTLD	64.9	59/48	PIB	V&Q	SUVR = 1.2	MMSE, CDR	NA	AD vs. FTLD
2011	Villemagne VL	Villemagne et al. (2011)	Australia	Prosp	Diagnostic value of florbetaben for AD, MCI, and OD	109	30 AD, 20 MCI, 32 NC, 11 FTLD, 7 DLB, 5 PD, 4 VD	71.1	64/45	Florbetaben	V&Q	SUVR = 1.4	NINCDS‐ADRDA, NINDS‐AIREN, MMSE, CDR, NBT	NA	AD vs. NC; AD vs. MCI
2012	Camus V	Camus et al. (2012)	France	Prosp	Diagnostic value of AV45 for AD and MCI	46	13 AD, 12 MCI, 21 NC	69	20/26	AV45	V&Q	SUVR = 1.122	NINCDS‐ADRDA, MMSE, DSM‐IV, FCSRT, NBT	NA	AD vs. NC
2012	Clark CM	Clark et al. (2012)	USA	Prosp	Diagnostic value of AV45 for AD	59	29 AD, 5 MCI, 12 NC, 13 non‐ADD	79.4	30/29	AV45	V&Q	SUVR = 1.10	Brain autopsy	6.6 months	AD vs. non‐ADD
2012	Jack CR Jr	Jack et al. (2012)	USA	Retro	Diagnostic value of MRI, FDG PET, and PIB PET for AD	492	42 AD, 450 NC	78.2	271/221	PIB	Q	SUVR = 1.5	NIA‐AA, MMSE, NBT, WAIS‐R, WMS‐R, AVLT, TMT, CFT, BNT	NA	AD vs. NC
2012	Mikhno A	Mikhno et al. (2012)	USA	Retro	Diagnostic value of MRI, FDG PET, and AV45 PET for AD and MCI	56	17 AD, 22 MCI, 17 NC	NR	NR	PIB	Q	^b^SVM classifier	NINCDS‐ADRDA, MMSE	NA	AD vs. NC; AD vs. MCI
2012	Newberg AB	Newberg et al. (2012)	USA	Retro	Comparing the diagnostic efficacy of AV45 and FGD for AD	40	19 AD, 21 NC	69.9	21/19	AV45	V	/	NINCDS‐ADRDA, MMSE	NA	AD vs. NC
2013	Brück A	Brück et al. (2013)	Finland	Prosp	Comparing the predictive efficacy of MRI, FDG PET, and PIB PET for MCI‐to‐AD conversion	29	17 cMCI, 12 sMCI	71.7	18/11	PIB	Q	SUVR = 1.57	MMSE, WLLT, WLRT	2 years	cMCI vs. sMCI
2013	Hatashita S and Yamasaki H	Hatashita and Yamasaki (2013)	Japan	Prosp	Comparing the predictive efficacy of FDG and PIB for MCI‐to‐AD conversion	68	30 cMCI, 38 sMCI	50–90	NR	PIB	V	/	NINCDS‐ADRDA, NIA‐AA, MMSE, CDR, WMS‐R, LM2	19.2 ± 7.1 years	cMCI vs. sMCI
2014	Beach TG	Beach et al. (2014)	USA	Prosp	Diagnostic value of AV45 for AD	919	618 AD, 301 non‐ADD	79	551/368	AV45	V	/	Brain autopsy	10.8 months	AD vs. non‐ADD
2014	Hatashita S	Hatashita et al. (2014)	Japan	Prosp	Comparing the diagnostic efficacy of FMM and PIB for AD	166	36 AD, 68 MCI, 62 NC	68.3	71/95	PIB; FMM	V	/	MMSE, CDR, WMS‐R, LM2	NA	AD vs. NC
2014	Kaneko N	Kaneko et al. (2014)	Japan	Retro	Diagnostic value of plasma amyloid‐related markers for AD	62	17 AD, 12 MCI, 33 NC	74.1	27/35	PIB	V	/	NIA‐AA, ADAS‐Jcog, MMSE, LM2, GDS	NA	AD vs. NC
2014	Mattsson N	Mattsson et al. (2014)	^a^USA, Canada	Prosp	Diagnostic value of CSF markers and AV45 PET for AD	511	118 AD, 59 cMCI, 165 sMCI, 169 NC	73.6	278/233	AV45	Q	SUVR = 1.11	NINCDS‐ADRDA, MMSE, CDR	2 years	AD vs. NC; cMCI vs. sMCI
2014	Saint‐Aubert L	Saint‐Aubert et al. (2014)	France	Prosp	Diagnostic value of AV45 for AD	39	22 AD, 17 NC	71.3	19/20	AV45	V&Q	SUVR = 1.28	MMSE, CDR, FCSRT	NA	AD vs. NC
2014	Trzepacz PT	Trzepacz et al. (2014)	^a^USA, Canada	Prosp	Comparing the predictive efficacy of MRI, FDG PET, and PIB PET for MCI‐to‐AD conversion	50	20 cMCI, 30 sMCI	74.7	33/17	PIB	V	/	NINCDS‐ADRDA, ADAS‐cog, MMSE, CDR, LM2	2 years	cMCI vs. sMCI
2014	Tzen KY	Tzen et al. (2014)	Taiwan	Retro	Diagnostic value of plasma Aβ and tau proteins for AD and its correlation with PIB PET	45	25 AD, 20 NC	65.4	20/25	PIB	Q	SUVR = 1.5	NIA‐AA, MMSE, CDR, NBT	NA	AD vs. NC
2015	Hosokawa C	Hosokawa et al. (2015)	Japan	Retro	Analyzing PIB's equivocal diagnostic findings for brain amyloid deposition	101	30 AD, 25 MCI, 19 NC, 8 LBD, 7 FTLD, 12 OD	69.5	44/57	PIB	V	/	NINCDS‐ADRDA, MMSE	NA	AD vs. non‐ADD
2015	Kerbage C	Kerbage et al. (2015)	USA	Retro	Correlation of Aβ deposition in the ocular lens with brain Aβ deposition revealed by AV45 PET	40	20 AD, 20 NC	74	22/18	AV45	V	/	NINCDS‐ADRDA, DSM‐IV, LM2	NA	AD vs. NC
2015	Li QX	Li et al. (2015)	Australia	Retro	Diagnostic value of Aβ PET for AD and correlation between CSF and SUVR	157	16 AD, 21 MCI, 120 NC	72.5	72/85	PIB; AV45; FMM	Q	SUVR = 1.50	MMSE, CDR		AD vs. NC; AD vs. MCI
2015	Schreiber S	Schreiber et al. (2015)	^a^USA, Canada	Prosp	Comparison of the diagnostic efficacy of AV45 with visual analysis and quantitative analysis for MCI‐to‐AD conversion	401	61 cMCI, 240 sMCI	71.6	219/182	AV45	V&Q	SUVR = 1.1	NINCDS‐ADRDA, MMSE, LM2	1.6 years	cMCI vs. sMCI
2015	Villeneuve S	Villeneuve et al. (2015)	USA	Prosp	Comparison of the diagnostic efficacy of quantitative analysis of different thresholds of PIB for AD	50	50 AD	69.8	33/17	PIB	Q	SUVR = 1.21	Brain autopsy	3.1 years	AD
2016	Chen X	Chen et al. (2016)	^a^USA, Canada	Prosp	Predictive value of FDG, PIB PET, and CSF biomarkers for MCI‐to‐AD conversion	82	34 AD, 48 MCI	74.4	60/22	PIB; AV45	Q	SUVR = 1.362	NINCDS‐ADRDA, ADAS‐cog, MMSE, CDR; follow‐up	96 months	cMCI vs. sMCI
2016	Dukart J	Dukart et al. (2016)	^a^USA, Canada	Prosp	Diagnostic value of MRI, FDG PET, AV45 PET for AD and predictive value for MCI‐to‐AD conversion	698	144 AD, 177 cMCI, 265 sMCI, 112 NC	74.8	416/282	AV45	Q	SUVR = 1.1	NINCDS‐ADRDA, ADAS, MMSE, RAVLT, GDS, FAQ	2 years	AD vs. NC; cMCI vs. sMCI
2016	Seo SW	Seo et al. (2017)	USA	Prosp	Diagnostic value of regional or global SUVR of PIB for AD	54	12 AD, 6 MCI, 2 NC, 33 FTLD, 1 VD	68.1	33/21	PIB	Q	SUVRglobal = 1.21	Brain autopsy	3.1 ± 1.9 years	AD vs. non‐ADD
2016	Wang MJ	Wang, Yi et al. (2016)	Korea	Retro	Diagnostic value of PIB PET combined with CSF for AD and the correlation between CSF and PIB PET	57	27 AD, 30 NC	65.3	30/27	PIB	Q	SUVR = 1.259	NIA‐AA, MMSE, CDR, NPI, GDS	NA	AD vs. NC
2016	Wang P	Wang, Chen et al. (2016)	^a^USA, Canada	Prosp	Predictive value of MRI, FDG PET, and AV45 PET for MCI‐to‐AD conversion	129	64 cMCI, 65 sMCI	72.3	74/55	AV45	Q	^b^Voxels‐analysis	ADAS‐cog, MMSE, CDR; Follow‐up	3 years	cMCI vs. sMCI
2016	Xu L	Xu et al. (2016)	^a^USA, Canada	Prosp	Predictive value of MRI, FDG PET, and AV45 PET for MCI‐to‐AD conversion	227	27 cMCI, 83 sMCI, 117 NC	75.3	141/86	AV45	Q	^b^ *α* in wmSRC = 0.6	MMSE, CDR	3 years	cMCI vs. sMCI
2016	Zwan MD	Zwan et al. (2016)	Several Countries	Retro	Diagnostic threshold for CSF based on brain Aβ deposits shown by PIB PET	433	195 AD, 98 MCI, 57 NC, 83 non‐ADD	64.6	256/177	PIB	V	/	MMSE, NPI	NA	AD vs. non‐ADD
2017	Ben Bouallegue F	Ben Bouallegue et al. (2017)	^a^USA, Canada	Prosp	Efficacy of Aβ PET and CSF in diagnosing AD and predicting MCI‐to‐AD conversion	677	124 AD, 301 MCI, 157 NC, 95 SMC	71.9	83/104	AV45	Q	SUVRcomp = 0.89	NINCDS‐ADRDA, ADAS‐cog, MMSE, CDR	22 ± 13 months	AD vs. NC
2017	Iaccarino L	Iaccarino et al. (2017)	Sweden	Retro	Efficacy of FDG and PIB in predicting MCI‐to‐AD conversion	30	14 cMCI, 16 sMCI	63.6	10/20	PIB	Q	SUVR = 1.41	MMSE, SWAIS, NBT	26.5 months	cMCI vs. sMCI
2017	Takahashi R	Takahashi et al. (2017)	^a^USA, Canada	Retro	Diagnostic value of FDG combined with AV45 for AD	324	143 AD, 181 NC	73.4	171/153	AV45	Q	^b^Voxels‐analysis	NINCDS‐ADRDA		AD vs. NC
2017	Zhang N	Zhang et al. (2017)	China	Retro	Correlation between relevant indicators in urine and brain Aβ deposition	30	20 AD, 10 MCI	66.3	9/21	PIB	Q	SUVR = 1.5	NINCDS‐ADRDA, MMSE, CDR, Petersen criteria, NPI	NA	AD vs. MCI
2018	Alvarez I	Alvarez et al. (2018)	Spain	Retro	Correlation of CSF biomarkers with Aβ PET	68	46 AD, 6 FTLD, 2 non‐ADD, 1 VD, 13 UD	62.9	27/41	AV45	V	/	IWG‐2 criteria, MMSE, Petersen criteria	NA	AD vs. non‐ADD
2018	Mielke MM	Mielke et al. (2018)	USA	Prosp	Diagnostic value of plasma phospho‐tau181 in AD and correlation of plasma phospho‐tau181 with tau‐ and Aβ PET	269	40 AD, 57 MCI, 172 NC	71.2	187/82	PIB	Q	SUVR = 1.42	NIA‐AA, MMSE, CDR, DSM‐IV	NA	AD vs. NC; AD vs. MCI
2018	Oliveira F	Oliveira et al. (2018)	Several Countries	Retro	Diagnostic value of PIB PET combined with CSF for AD and the relationship between CSF and PIB retention	243	122 AD, 81 MCI, 13 NC, 20 FTLD, 7 VD	64.6	105/138	PIB	V	/	NINCDS‐ADRDA, NINDS‐AIREN, Petersen criteria, Neary criteria	NA	AD vs. NC; AD vs. MCI
2019	La Joie R	La Joie et al. (2019)	USA	Retro	Diagnosis of AD by PIB PET and using the ^b^Centiloid method	179	63 AD, 27 MCI, 22 NC, 67 non‐ADD	73	116/63	PIB	Q	^b^CL = 24.4	Brain autopsy	3.3 years	AD vs. NC
2019	Li WW	Hajiramezanali et al. (2019)	China	Retro	Correlation of brain Aβ deposition in AD with blood Aβ levels as shown by PIB PET	84	53 AD, 22 MCI, 9 NC	66.7	41/43	PIB	V	/	NINCDS‐ADRDA, MMSE, CDR, Petersen criteria, DSM‐IV, MoCA	NA	AD vs. NC; AD vs. MCI
2019	Ottoy J	Ottoy et al. (2019)	Belgium	Retro	Correlation between CSF, MRI, Aβ PET and MCI‐to‐AD conversion at baseline	78	16 AD, 49 MCI, 13 NC	72	41/37	AV45	V&Q	SUVR = 1.203	NIA‐AA, MMSE, RBANS	415 ± 18 days	AD vs. NC; AD vs. MCI
2019	Park JC	Park et al. (2019)	Korea	Prosp	Predictive value of blood biomarkers for brain Aβ deposition	254	40 AD, 107 MCI, 107 NC	71.5	89/165	PIB	Q	SUVR = 1.4	MMSE, CDR	NA	AD vs. NC; AD vs. MCI
2019	Peretti DE	Peretti et al. (2019)	Belgium	Prosp	Diagnostic value of dynamic PIB of rCBF images for AD	52	15 AD, 21 MCI, 16 NC	66.6	35/17	PIB	Q	^b^PET‐score = 2.08	NIA‐AA, MMSE	NA	AD vs. NC; AD vs. MCI
2020	Chanisa C	Chanisa et al. (2021)	Thailand	Prosp	Diagnostic value of PIB for AD	40	16 AD, 24 NC	60.1	18/22	PIB	Q	SUVR = 1.5	NIA‐AA, MMSE, CDR, MoCA	NA	AD vs. NC
2021	Kitajima K	Kitajima et al. (2021)	Japan	Retro	Diagnostic value of PIB for AD and MCI	26	7 AD, 15 MCI, 1 NC, 1 VD, 2 FTLD	78.5	5/21	PIB	V&Q	SUVR = 1.5	NINCDS‐ADRDA, MMSE, MoCA, FAB	NA	AD vs. MCI; AD vs. non‐ADD
2021	Lesman‐Segev OH	Lesman‐Segev et al. (2021)	USA	Prosp	Comparing the diagnostic efficacy of PIB and FDG for AD	101	32 AD, 56 FTLD, 13 mixed AD/FTLD	67.2	60/41	PIB	V	/	Brain autopsy	4.4 years	AD vs. FTLD
2021	Park JC	Park et al. (2021)	Korea	Prosp	Predictive value of plasma samples for brain Aβ deposition	300	64 AD, 87 MCI, 149 NC	71.1	113/187	PIB	Q	SUVR = 1.4	NIA‐AA, MMSE, CDR, DSM‐IV	NA	AD vs. NC; AD vs. MCI

^Note:^ *NINCDS‐ADRDA/NINDS‐AIREN* the criteria for probable AD. *NIA‐AA* the criteria for diagnosing AD. *Neary criteria* the criteria for FLTD. *Petersen criteria* the criteria for MCI.

**^Abbreviations:^ :**AD, Alzheimer's disease; ADAS‐cog, AD assessment scale‐cognitive subscale; ADAS‐Jcog, ADAS‐Jcog (Japanese version); AVLT, auditory verbal learning test; Aβ, amyloid‐β; BNT, Boston Naming Test; CDR, clinical dementia rating scale; CFT, category fluency test; cMCI, MCI converting to AD; DLB, dementia with Lewy bodies; DSM‐IV, Diagnostic and Statistical Manual of Mental Disorders (fourth edition); FAB, Frontal Assessment Battery; FAQ, Functional Activities Questionnaire; FCSRT, Free and Cued Selective Reminding Test; FTLD, frontotemporal dementia; GDS, Geriatric Depression Scale; IWG‐2, criteria the International Working Group for new research criteria for the diagnosis of Alzheimer's disease; LM2, logical memory II from the WMS‐R; MCI, mild cognitive impairment; MMSE, mini‐mental state examination; MoCA, Montreal Cognitive Assessment; NBT, neuropsychological battery test; NC, normal controls; Non‐ADD, other non‐AD dementia; NPI, neuropsychiatric inventory; OD, other mental disorders; PD, Parkinson disease; RAVLT, Rey Auditory Verbal Learning Test; RBANS, the Repeatable Battery for the Assessment of Neuropsychological Status; SMC, significant memory complaint; sMCI, stable MCI; SUVR, standardized uptake value ratio; SWAIS, Swedish versions of the Wechsler Adult Intelligence Scale; TMT, Trail Making Test; UD, uncertain diagnosis; VD, vascular dementia; WAIS‐R, Wechsler Adult Intelligence Scale‐Revised; WLLT, word list learning test score; WLRT, word list recall test score; WMS‐imd, Wechsler Logical Memory Scale immediate recall test; WMS‐R, Wechsler Memory Scale‐Revised.

^a^ Using the ADNI database.

^b^ Using specific quantitative analysis methods; *Ref* reference; *N of pts* number of patients; *NR* not report; *NA* not available; *Retro* retrospective; *Prosp* prospective; *CSF* cerebrospinal fluid; *rCBF* regional cerebral blood flow; *V* visual analysis; *Q qualitative analysis;* *PIB* ^11^C‐PIB; *AV45* ^18^F‐AV45; *FMM* ^18^F‐FMM; *Florbetaben* ^18^F‐florbetaben.

### 3.2 Methodological qualitative analysis

**FIGURE 3:**
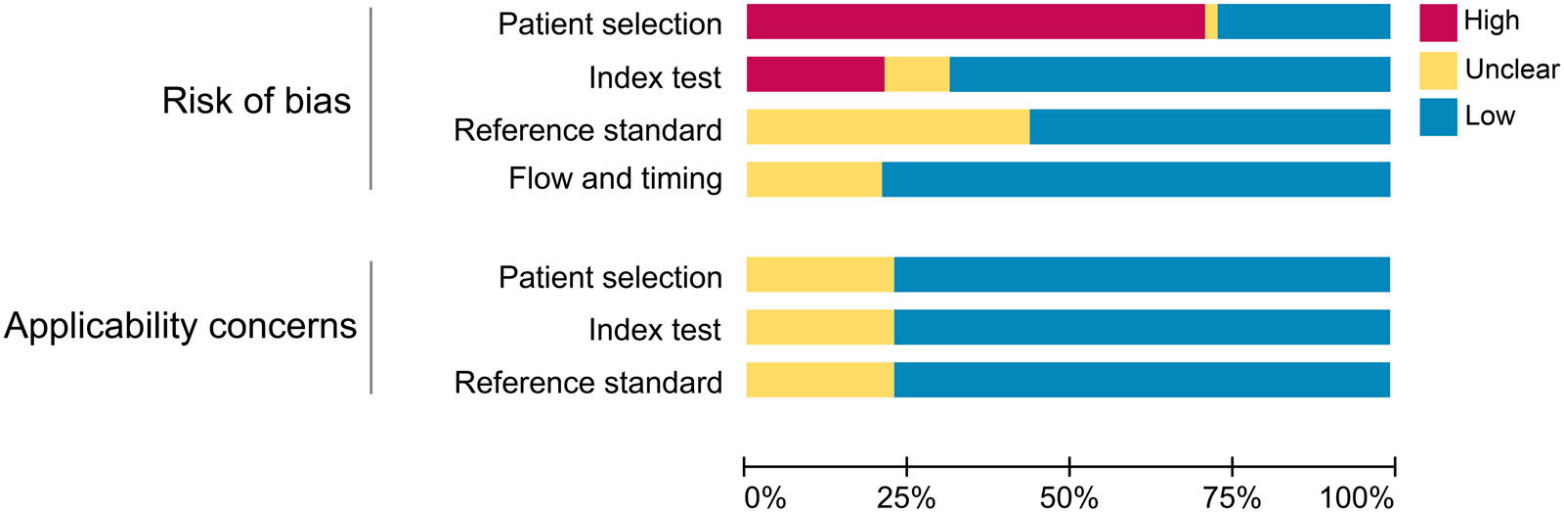
Overall assessment of the methodological quality of the 48 studies, including evaluation of risk bias and evaluation of applicability concerns

The risk bias and clinical applicability assessment results for each study are shown in Table 2. Thirty‐four studies in patient selection were assessed as high risk because they were case‐control studies. Ten studies were assessed as high risk for the index test because they were unblinded, and the threshold was set after the examination. Overall, studies with high‐risk bias in patient selection were upward of 50%; studies with high‐risk bias in index tests were approximately 20%, and uncertain risk bias was approximately 10%; studies with uncertain risk bias in reference standards were approximately 40%, and studies with uncertain risk bias in flow and timing were approximately 20%. For clinical applicability concerns, no study was evaluated as highly inapplicable, but more than 20% of the studies were evaluated as having uncertain concerns (Figure 3).

**TABLE 2:** Quality assessment of the included studies based on Quality Assessment of Diagnostic Accuracy Studies‐2 (QUADAS‐2)

	Risk of bias				Applicability concerns		
Study	Patient selection	Index test	Reference standard	Flow and timing	Patient selection	Index test	Reference standard
2007 Ng S	High	Low	Low	Low	Low	Low	Low
2010 Tolboom N	High	Low	Low	Low	Low	Low	Low
2010 Vandenberghe R	High	Low	Low	Low	Low	Low	Low
2011 Fleisher AS	High	Low	Low	Low	Low	Low	Low
2011 Rabinovici GD	Low	Low	Low	Low	Low	Low	Low
2011 Villemagne VL	High	Low	Low	Low	Low	Low	Low
2012 Camus V	High	Low	Low	Low	Low	Low	Low
2012 Clark CM	Low	Low	Low	Low	Low	Low	Low
2012 Jack CR Jr	High	High	Unclear	Low	Low	Low	Low
2012 Mikhno A	High	High	Unclear	Low	Low	Low	Low
2012 Newberg AB	High	Low	Low	Low	Low	Low	Low
2013 Brück A	Low	High	Unclear	Low	Low	Low	Low
2013 Hatashita S	Low	Unclear	Unclear	Low	Low	Low	Low
2014 Beach TG	Low	Low	Low	Low	Low	Low	Low
2014 Hatashita S	Unclear	Unclear	Unclear	Low	Low	Low	Low
2014 Kaneko N	High	Low	Low	Unclear	Unclear	Unclear	Unclear
2014 Mattsson N	High	Low	Unclear	Low	Low	Low	Low
2014 Saint‐Aubert L	High	Low	Low	Low	Low	Low	Low
2014 Trzepacz PT	Low	Unclear	Unclear	Low	Low	Low	Low
2014 Tzen KY	High	Low	Low	Unclear	Unclear	Unclear	Unclear
2015 Hosokawa C	Low	Low	Low	Unclear	Unclear	Unclear	Unclear
2015 Kerbage C	High	Low	Low	Unclear	Unclear	Unclear	Unclear
2015 Li QX	High	Low	Low	Low	Low	Low	Low
2015 Schreiber S	Low	Low	Low	Low	Low	Low	Low
2015 Villeneuve S	Low	Low	Low	Low	Low	Low	Low
2016 Chen X	High	High	Unclear	Low	Low	Low	Low
2016 Dukart J	High	Low	Unclear	Low	Low	Low	Low
2016 Seo SW	Low	Low	Low	Low	Low	Low	Low
2016 Wang MJ	High	High	Unclear	Low	Low	Low	Low
2016 Wang P	Low	Low	Low	Low	Low	Low	Low
2016 Xu L	High	High	Unclear	Low	Low	Low	Low
2016 Zwan MD	High	Low	Unclear	Unclear	Unclear	Unclear	Unclear
2017 Ben Bouallegue F	High	High	Unclear	Low	Low	Low	Low
2017 Iaccarino L	High	Low	Low	Low	Low	Low	Low
2017 Takahashi R	High	High	Unclear	Low	Low	Low	Low
2017 Zhang N	High	Low	Low	Unclear	Unclear	Unclear	Unclear
2018 Alvarez I	Low	Unclear	Unclear	Unclear	Unclear	Unclear	Unclear
2018 Mielke MM	High	Low	Unclear	Unclear	Unclear	Unclear	Unclear
2018 Oliveira F	High	Unclear	Unclear	Low	Low	Low	Low
2019 La Joie R	High	Low	Low	Low	Low	Low	Low
2019 Li WW	High	Low	Low	Unclear	Unclear	Unclear	Unclear
2019 Ottoy J	High	Low	Unclear	Low	Low	Low	Low
2019 Park JC	High	Low	Unclear	Unclear	Unclear	Unclear	Unclear
2019 Peretti DE	High	High	Unclear	Low	Low	Low	Low
2020 Chanisa C	High	High	Unclear	Low	Low	Low	Low
2021 Kitajima K	High	Low	Low	Low	Low	Low	Low
2021 Lesman‐Segev OH	Low	Low	Low	Low	Low	Low	Low
2021 Park JC	High	Low	Low	Low	Unclear	Unclear	Unclear

### 3.3 Pooled diagnostic performance of Aβ PET

**FIGURE 4:**
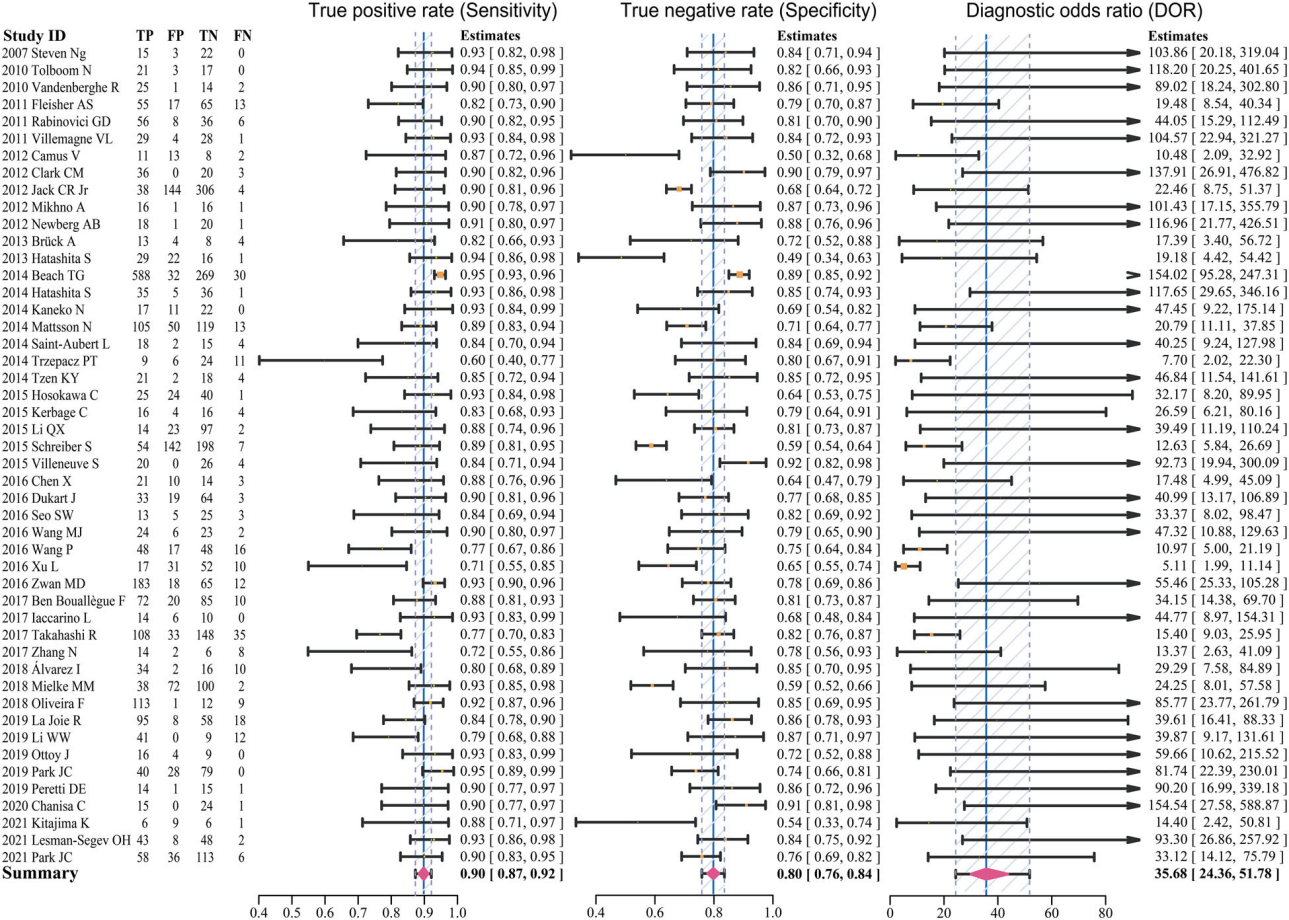
Forest plots of pooled sensitivity, specificity, and DOR for the included studies

**FIGURE 5:**
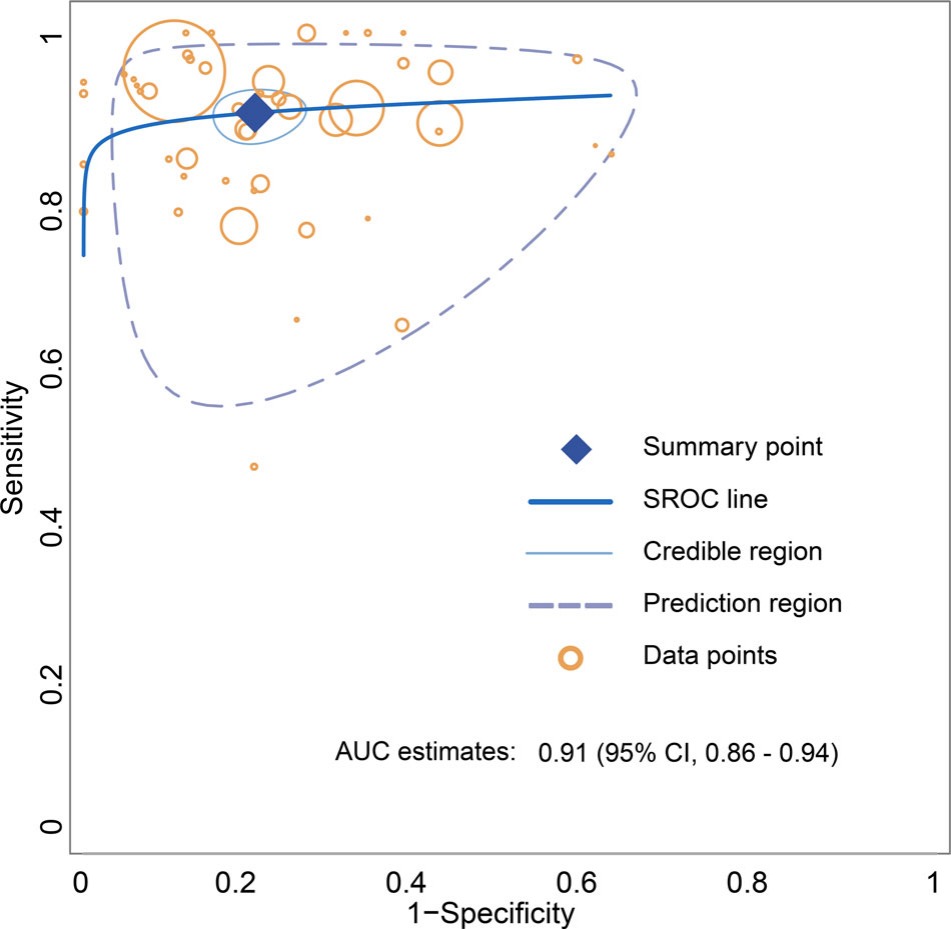
Summary ROC curve for the overall performance assessment of amyloid‐β (Aβ) PET for the diagnosis of Alzheimer's disease (AD)

The results of our meta‐analysis of 48 studies are shown in Figure 4. The studies that performed the overall pooled assessment included 29 AD versus NC, 2 AD versus MCI, 2 AD versus FTLD, 6 AD versus non‐ADD, 8 cMCI versus sMCI, and 1 AD only. The pooled sensitivity, specificity, DOR, and AUC of Aβ PET for AD diagnosis were 0.90 (95% CI 0.87–0.92), 0.80 (95% CI 0.76–0.84), 35.68 (95% CI 24.36–51.78), and 0.91 (95% CI 0.86–0.94), respectively. The trend of the SROC curve was close to the upper left corner, and the distribution of individual studies was primarily concentrated in the upper left corner, all of which suggested that the overall diagnostic performance of Aβ PET was favorable. The dispersion of individual studies shown in the SROC curve was not significant and was mostly concentrated within the confidence interval, thus potentially suggesting that the heterogeneity between studies was within an acceptable range (Figure 5).

We compared the pooled diagnostic performance of the AD versus NC (Figure S1), AD versus MCI (Figure S2), and cMCI versus sMCI (Figure S3) groups, respectively. The pooled sensitivity of Aβ PET for diagnosing AD (sensitivity: 0.91, 95% CI 0.88–0.93) was higher than that of cMCI (sensitivity: 0.84, 95% CI 0.74–0.92). Aβ PET had the highest pooled specificity for differentiating NC (specificity: 0.81, 95% CI 0.77–0.86) and performed poorly in differentiating MCI (specificity: 0.49, 95% CI 0.41–0.58). The area under the diagnostic SROC curve for the three population groups of AD versus NC, AD versus MCI, and cMCI versus sMCI was 0.93 (95% CI 0.89–0.95), 0.83 (95% CI 0.80–0.95), and 0.78 (95% CI 0.73–0.92), respectively. The diagnostic pooled sensitivity (0.91 vs. 0.86), specificity (0.80 vs. 0.78), DOR (45.66 vs. 22.12), and AUC (0.93 vs. 0.86) of ^11^C‐PIB PET (Figure S4) were higher than ^18^F‐AV45 PET (Figure S5). In addition, the pooled sensitivity (0.92 vs. 0.90), specificity (0.85 vs. 0.83), DOR (78.74 vs. 49.56), and AUC (0.94 vs. 0.92) of visual assessment (Figure S6) were higher than those of quantitative analysis (Figure S7). The pooled estimates of these detailed comparisons are shown in Table 3.

**TABLE 3:** Comparison of diagnostic performance between subgroups

Data‐type	Pooled sensitivity (95% CI)	Pooled specificity (95% CI)	Pooled diagnostic OR (95% CI)	Pooled AUC estimates (95% CI)
Population groups				
AD vs. NC	0.91 (0.88–0.93)	0.81 (0.77–0.86)	45.32 (29.12–71.29)	0.93 (0.89–0.95)
AD vs. MCI	0.90 (0.85–0.95)	0.49 (0.41–0.58)	9.88 (5.30–18.42)	0.83 (0.80–0.95)
cMCI vs. sMCI	0.84 (0.74–0.92)	0.62 (0.56–0.68)	9.26 (4.44–17.66)	0.78 (0.73–0.92)
Different radiotracers				
^11^C‐PIB	0.91 (0.88–0.94)	0.80 (0.75–0.85)	45.66 (25.98–74.25)	0.93 (0.90–0.96)
^18^F‐AV45	0.86 (0.81–0.90)	0.78 (0.70–0.84)	22.12 (12.34–39.26)	0.86 (0.64–0.92)
Diagnostic methods				
Visual analysis	0.92 (0.88–0.96)	0.85 (0.77–0.91)	78.74 (31.97–176.49)	0.94 (0.89–0.97)
Quantitative analysis	0.90 (0.87–0.93)	0.83 (0.79–0.88)	49.56 (30.35–80.68)	0.92 (0.89–0.95)

Abbreviations: AD, Alzheimer's disease; MCI, mild cognitive impairment; NC, normal controls.

### 3.4 Publication bias

**FIGURE 6:**
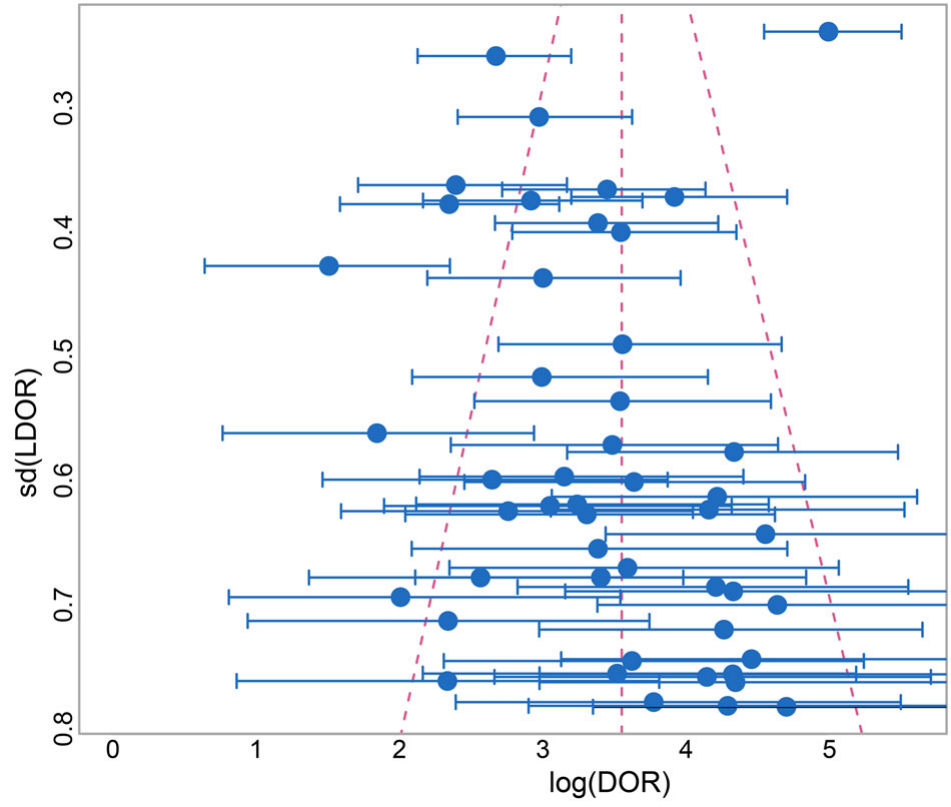
Funnel plot for assessing publication bias and heterogeneity

We made an overall assessment of publication bias and heterogeneity of the studies after visual analysis of the funnel plot (Figure 6). Overall, the studies were roughly evenly distributed on both sides of the log diagnostic OR estimates, indicating no significant publication bias. In addition, a large proportion of studies were clustered at the bottom of the funnel plot, reflecting that we included many small samples of studies, which may have led to less stability in the pooled estimates. A small portion of studies at the top and upper middle of the funnel plot is scattered outside the 95% confidence interval, whereas the majority of studies are within the 95% confidence interval; therefore, the heterogeneity among studies is moderate and within an acceptable range.

## 4 DISCUSSIONS

Over the past 30 years, the most widely used diagnostic guideline for AD is the NINCDS‐ADRDA diagnostic criteria, the main diagnostic criteria of which are (1) dementia identified by clinical examination and cognitive scales, (2) two or more cognitive deficits with progressive deterioration, (3) no impairment of consciousness, (4) onset at age 40–90 years, and (5) exclusion of other systemic and brain diseases that cause progressive memory and cognitive impairment (Mckhann et al., 1984). As research on AD has become more advanced, new insights into the sequence of onset and progression of AD have emerged. The guidelines for the management of AD published in 2011 (NIA‐AA criteria) focus on illustrating AD as a continuum involving pathophysiological changes to the progressive appearance of clinical symptoms (Albert et al., 2011; Jack et al., 2011; Sperling et al., 2011). The NIA‐AA guidelines consider Aβ deposition as the first step in the progression of AD, followed by downstream changes in a range of indicators of neurodegeneration and then by a slight decline in cognitive function. The biomarkers used to diagnose AD are therefore divided into two categories: (1) reflecting Aβ deposition, such as Aβ42 in cerebrospinal fluid (CSF) or Aβ PET; and (2) reflecting neuronal damage, such as CSF tau/p‐Tau, hippocampal or medial temporal lobe atrophy on MRI, and glucose hypometabolism on PET or SPECT. We pooled studies from 2007 to 2021 on Aβ PET for AD diagnosis and prediction of MCI conversion to AD. Based on the results of our meta‐analysis, we evaluate the diagnostic and predictive performance of Aβ PET and comprehensively discuss how to correctly and effectively maximize the diagnostic accuracy in clinical applications and the problems and challenges currently faced in clinical work. Our pooled estimates suggest that Aβ PET is highly sensitive in determining AD and possesses favorable diagnostic efficacy overall but performs with average specificity, which is similar to the meta‐analysis by Morris et al. (2016).

First, the slightly lower specificity in distinguishing AD from NC is due to the false positives in cognitively NC. Aβ deposition was present in 20%–30% of cognitively normal elderly subjects (Dahmer et al., 1991). As mentioned above, pathological Aβ deposition occurs prior to cognitive impairment, and it remains to be confirmed in longitudinal studies whether healthy controls with ^11^C‐PIB retention are false positives or preclinical stages of AD. The accuracy of ^11^C‐PIB in diagnosing AD is generally less affected by age, but the older the population, the more likely it is to have false positives. The most likely reason is that non‐demented older adults over 75‐year‐old have multiple diffuse Aβ plaques in the neocortical areas, making the ^11^C‐PIB PET less specific (Ng et al., 2007; Price & Morris, 1999). In Steven's study, the diagnostic accuracy of either ^18^F‐FDG or ^11^C‐PIB significantly decreased with age in differentiating between AD and healthy controls (Ng et al., 2007). Similarly, the results of the ^18^F‐AV45 PET study showed that the retention of tracers in healthy controls increased with age (Fleisher, 2011).

Aβ PET had the lowest specificity (below 60%) when comparing AD and MCI. It is similar to the pooled results of Morris et al. (2016), who concluded that the specificity was significantly lower when MCI patients were included in the healthy control group. MCI is generally divided into two categories, amnestic MCI, which is dominated by memory decline, and non‐amnestic MCI, which is dominated by the decline in attention, executive ability, visual discrimination, and language skills. Amnestic MCI has been reported to have a higher probability of conversion to dementia, especially AD dementia, than non‐amnestic MCI (Busse et al., 2006). Kitajima's study showed that 60% of MCI patients were positive for ^11^C‐PIB. Seventy‐five percent of ^11^C‐PIB‐positive MCI were amnestic MCI, whereas none of the non‐amnestic MCI patients had ^11^C‐PIB uptake, that is, no evidence of Aβ deposition (Kitajima et al., 2021). Eighty‐two percent of MCI patients with Aβ deposits were diagnosed with AD at the 3‐year follow‐up period, whereas 7% of MCI patients without Aβ deposits converted to AD (Okello et al., 2009). Therefore, when AD and MCI are mixed for differential diagnosis, the high uptake of Aβ tracer in patients with potentially progressive MCI could explain the high false positive and the low specificity.

On the other hand, Aβ PET was slightly more sensitive and less specific in predicting the conversion of MCI to AD. The predictive performance of Aβ PET in the study by Zhang et al. (2012) was essentially similar to ours, with their sensitivity (93.5%) slightly higher and specificity (56.2%) slightly lower than our results. His study addressed the hypothesis that neurofibrillary tangles may occur prior to Aβ plaques during the period of MCI converting to AD, whereas Aβ plaques are relatively rare in the early stages, which may explain ^11^C‐PIB's low specificity and sensitivity for predicting cMCI. The study comparing ^18^F‐FDG and ^11^C‐PIB found that the conversion rate of MCI was 100% in ^18^F‐FDG‐positive (characteristically hypo‐metabolic) subjects compared to 70% in ^11^C‐PIB‐positive subjects. However, an additional 3/14 cMCI patients showed positive ^11^C‐PIB and negative ^18^F‐FDG, suggesting that patients with MCI progressing to AD may be detected earlier with ^11^C‐PIB PET than with ^18^F‐FDG PET (Iaccarino et al., 2017). As mentioned above, Aβ deposition in the brain is progressive with age, but such Aβ positivity does not progress to dementia or cognitive decline. Therefore, the increased Aβ load in MCI patients does not provide sufficient confidence to diagnose MCI progression to AD. Several studies combining three imaging data modalities, that is, morphology, glucose metabolism, and Aβ load, on predicting whether patients with MCI will progress to AD have shown that most MCI can be accurately classified (Chen et al., 2016; Wang, Chen et al., 2016; Xu et al., 2016). It suggests that the diagnostic information provided by multimodal imaging of Aβ PET combined with ^18^F‐FDG PET or MRI may maximize diagnostic accuracy, especially in patients with an equivocal diagnosis of MCI.

Regarding comparing the radiotracer of ^11^C‐PIB and ^18^F‐AV45, our results suggest that ^11^C‐PIB has better sensitivity and specificity than ^18^F‐AV45. Previous meta‐analysis results showed no difference in diagnostic sensitivity and specificity between the different radiotracers. However, the authors considered the possibility of including too few studies to show a difference (Morris et al., 2016). ^11^C‐PIB has a higher rate of cortical retention than ^18^F‐AV45, suggesting that ^11^C‐PIB PET imaging may be more sensitive in showing lesions (Landau et al., 2014). There is a low rate of clinical use of ^18^F‐florbetaben and even fewer studies comparing it with other radiotracers. Although the cortical distribution of ^18^F‐florbetaben is almost identical to that of ^11^C‐PIB, the degree of binding to Aβ is lower. Compared to the controls, patients in the AD group utilizing ^18^F‐florbetaben PET imaging had a 53% higher standardized uptake value ratio (SUVR) in the neocortical area. However, when ^11^C‐PIB was utilized, patients in the AD group had 60%–70% higher SUVR in the neocortical area than controls (Villemagne et al., 2011). There are also studies comparing the retention of different radiotracers in the white matter area. It was shown that ^18^F‐FMM had the highest white matter retention. However, the rate of ^18^F‐FMM and ^11^C‐PIB uptake was similar in all neocortical areas, rendering ^18^F‐FMM comparable to ^11^C‐PIB for diagnosing AD (Vandenberghe et al., 2010). A slightly higher degree of white matter retention was observed with ^11^C‐PIB than with ^18^F‐AV45, whereas the difference was practically negligible (Landau et al., 2014). Only when using white matter as the reference area, can the presence of higher retention of radiotracers in white matter areas affect visual judgments and quantitative measures of cortical retention. Notably, the newly emerged radiotracer ^18^F‐NAV4694 has a high affinity for brain Aβ plaques. In vitro experiments revealed that ^18^F‐NAV4694 has the same high affinity for Aβ deposits and showed selective labeling of Aβ in the cortex of the postmortem human brain (Jureus et al., 2010). Moreover, in a comparative study between ^18^F‐NAV4694 and ^11^C‐PiB, ^18^F‐NAV4694 binds Aβ with slightly higher affinity than ^11^C‐PIB. Meanwhile, ^18^F‐NAV4694 was found to have better stability and less variability in Aβ binding in young controls. The researchers speculate that this may be because ^18^F (109 min) has a longer half‐life than ^11^C (20 min) resulting in ^18^F‐NAV4694 producing a higher counts rate and better images during scanning (Rowe et al., 2016). In conclusion, ^18^F‐NAV4694 is probably the most accurate and convenient radiotracer available for assessing Aβ deposition in the brain.

Furthermore, we found that visual analysis could yield better diagnostic outcomes than quantitative analysis, although the difference was very small. The meta‐analysis by Elizabeth Morris suggests no difference between visual and quantitative assessment (Morris et al., 2016). Because of the low levels of cerebellar Aβ plaques found in autopsy pathology in AD patients, most studies use cerebellar cortical areas as a reference to determine the retention of ^11^C‐PIB in the region of interest, both in visual and quantitative assessments (Svedberg et al., 2009). The results of visual interpretation often depend on the clinical experience of the observer. In contrast, the diagnostic accuracy of quantitative analysis often depends on the appropriate threshold or the diagnostic model formula. Our pooled results do not reflect the true picture. Nine studies simultaneously made direct comparisons between quantitative and visual analyses (Camus et al., 2012; Clark et al., 2012; Ng et al., 2007; Rabinovici et al., 2011; Saint‐Aubert et al., 2014; Schreiber et al., 2015; Tolboom et al., 2010; Vandenberghe et al., 2010; Villemagne et al., 2011). Five studies showed that quantitative assessment was superior to visual assessment, three showed that visual assessment was superior to quantitative assessment, and one showed that both assessments were equally effective. Of particular interest is the study by Camus V, which showed a low specificity of 38.1% for the visual assessment and high specificity of 90.5% for the quantitative assessment. He attributed the possible reasons for this to the low spatial resolution of the reconstructed images and the presence of Aβ deposits in the group of NC (Camus et al., 2012). Early on, researchers referred to visual assessment as a diagnostic approach that does not require extensive training to make accurate diagnoses and has a very high consistency of results between interpreters (Ng et al., 2007). However, the truth is that visual assessment is subject to more influences, and the reliability of judgments between interpreters may require a great deal of learning and experience. First, focal and asymmetric ^18^F‐AV45 or ^11^C‐PIB retention may produce a positive visual assessment and a negative quantitative analysis of Aβ deposition. Further, when increased white matter retention is present, it is more likely to affect the results of visual analysis. In quantitative analysis, the average global cortical SUVR threshold ranged from 1.2 to 1.5, but setting the global SUVR threshold higher than 1.4 was thought to miss many AD patients (Villeneuve et al., 2015). Chanisa et al. (2021) mentioned that Aβ plaques tend to spread from the cerebral cortex to the cingulate and precuneus regions, whereas they also found the highest ^11^C‐PIB deposition in the anterior and posterior cingulate gyri. His study showed that making a diagnosis based on local SUVR achieved higher sensitivity and specificity than global SUVR, and that the cutoff values of regional SUVR (1.46–1.81) were higher than global. Previously, Seo et al. (2017) demonstrated by autopsy that there was no difference in the accuracy of peak regional and global SUVR of ^11^C‐PIB in predicting pathological Aβ load. Anyway, more studies are needed to reveal the answer to the question of whether global or regional SUVR measurements are suitable as diagnostic criteria. Moreover, by utilizing more advanced analysis methods, including voxel analysis, Gaussian mixture models, and cluster analysis, some studies obtained lower optimal SUVR thresholds. The researchers extracted considerable PET image features (90–1000 features) or combined them with clinical features for analysis, all of which resulted in accurate classification results (Oliveira et al., 2018; Xu et al., 2016). As nice as machine learning is, the number of redundant features tends to cause overfitting; therefore, proper dimensionality reduction to extract more sensitive features is the key to building a more stable and effective prediction model. The most important point that should not be overlooked is the variation in the results provided by each research center due to the unique affinity and kinetic properties of each tracer, the inconsistency of the interpreters’ approach to image analysis (different cortical regions of interest, different reference areas, different cutoff values, etc.), and the different technical factors of image acquisition (duration after tracer injection, acquisition duration, image reconstruction algorithms, etc.). The lack of standardized quantitative results, resulting in multiple cutoff values for diagnostic and prognostic determinations per study center, may hinder comparisons of studies across centers and limit comparisons of the relative effectiveness of current therapies directed at reducing Aβ burden. The Centiloid method standardizes Aβ PET imaging measurements by scaling the ^11^C‐PIB PET imaging measurements (SUVR) of NC and AD patients into Centiloid (CL) units by ratio (Klunk et al., 2015). Further, numerous studies provide new conversion equations for converting SUVR values for various ^18^F‐amylose tracers to CL units (Bourgeat et al., 2018; Rowe et al., 2016). The Centiloid method provides cutoff values for all Aβ PET and provides clinicians with valuable diagnostic and prognostic data.

According to our analysis, Aβ PET can accurately diagnose normal and AD patients but cannot distinguish well between AD and MCI patients. The overall performance of Aβ PET in determining the conversion of MCI to AD was very average. In addition to the analysis of the diagnostic results we pooled, several other findings help us understand the significance of these differences. ApoE ε4 is one of the most prominent genotypes in AD development and impacts the levels of other biomarkers, such as Aβ42 in CSF. The probability of carrying the gene of ApoE ε4 is significantly higher in MCI and AD patients than in normal subjects (Lautner et al., 2014). The average cortical SUVR was higher in normal subjects who were ApoE ε4 gene carriers than in noncarriers; similarly, the SUVR was higher in APOE ε4‐carrying AD patients than in noncarriers (Fleisher, 2011). Therefore, it is highly likely that Aβ PET is more sensitive in diagnosing AD patients carrying the ApoE ε4 allele. In our clinical work, we should pay more attention to those APOE ε4 carriers who present with positive Aβ PET images but have atypical clinical manifestations and be cautious in making the diagnosis of exclusion of AD. Several distinctive findings may also offer the potential to improve diagnostic efficacy. Kerbage et al. (2015) found a significant correlation between the concentration of Aβ in the lens of the eye and SUVR values of Aβ PET images in the brain, suggesting the possibility of evaluating brain Aβ load or even diagnosing AD by eye Aβ content. Zhang et al. (2017) mentioned a high correlation between AD7c‐NTP levels in urine and Aβ load in the brain on ^11^C‐PIB PET imaging. The positive predictive value of AD7c‐NTP in urine for predicting Aβ deposition in AD and MCI was 91.7%, and the negative predictive value was 72.2%. In addition, we paid slight attention to the time parameters of PET image acquisition. Duration after ^11^C‐PIB injection to PET image acquisition may vary, but most studies range from 40 to 90 min. Ng et al. (2007) demonstrated by dynamic scanning that the result of the visual analysis of PET images acquired at 30 min postinjection of ^11^C‐PIB was practically the same as the quantitative analysis of PET images acquired at 90 min postinjection of ^11^C‐PIB. It may be suggested that brain PET images acquired after 30 min postinjection of ^11^C‐PIB meet the demands of visual diagnosis.

In recent years, several completely different mechanisms of AD pathogenesis have been proposed from the mainstream Aβ cascade theory. Lee et al. (2022) proposed that neuronal cell death occurs first, followed by extracellular Aβ plaque formation. Aβ plaques are the remains of dead neuronal cells mixed with β amyloid. In addition, by using SUVR values of Aβ PET to reflect the load of insoluble β amyloid in the brain compared to the content of soluble Aβ42 in CSF, researchers found that higher levels of soluble Aβ42 were associated with better neuropsychological function and larger hippocampal volumes (Sturchio et al., 2021). Therefore, soluble Aβ42 levels may be a more effective response to cognitive impairment than Aβ deposition. To date, many anti‐Aβ drugs have been developed that effectively reduce Aβ deposits but most have failed to stop cognitive degeneration or slow the progression (Avgerinos et al., 2021). These aforementioned findings may be evidence that does not support the mainstream hypothesis of Aβ toxicity. It is worth highlighting that aducanumab, which targets anti‐Aβ, has been approved by the FDA for marketing as a drug for treating AD. Some studies have shown that aducanumab benefits patients with AD, but its efficacy and safety still need further validation (Alexander et al., 2021; Tolar et al., 2020). The significant role of Aβ PET is to monitor the dynamics of Aβ load in the brain continuously and to establish a correlation with the patient's condition, thus providing a comprehensive assessment of drug efficacy.

Finally, the limitations of our study need to be mentioned. First, there is no doubt that the AD diagnosis by brain autopsy or brain biopsy is more reliable than any mental status examination and neuropsychological testing battery. Nevertheless, the reference standard for most of our included studies was the clinical diagnosis of mental status examinations. The clinical diagnosis of AD may be influenced to some extent by the subjectivity of the patient or physician and may have a certain probability of deviation from the true picture. More than 10% of clinically diagnosed AD lack pathological features; overall, 19.1% of clinically diagnosed AD have lower levels of ^18^F‐AV45 uptake than pathologically diagnosed AD, and 14.7% of clinically diagnosed AD patients do not have a retention of ^18^F‐AV45 (Fleisher, 2011; Ranginwala et al., 2008). As a result, those studies that used the clinical mental status examination as a reference standard had a significant impact on the assessment of the diagnostic efficacy of Aβ PET, greatly likely increasing the false positive and false negative rates. An additional pitfall is that, as shown in Figure 6, we included many studies with small samples, which may portend low stability of our results.

## 5 CONCLUSIONS

Overall, ^11^C‐PIB and ^18^F‐AV45 PET have high sensitivity and specificity for diagnosing AD, especially in distinguishing AD from healthy individuals. Aβ PET has slightly lower diagnostic efficacy in predicting the progression of MCI to AD but can provide highly valuable prognostic information for clinical purposes. The specificity of Aβ PET to distinguish AD from MCI is low, and the impact of progressive MCI should be considered in clinical applications. The diagnostic specificity of Aβ PET will be effectively improved when combined with the glucose metabolic features of brain ^18^F‐FDG PET images or the morphological manifestations of MRI.

## AUTHOR CONTRIBUTIONS

Long Sun provided the ideas for this review, and Dan Ruan completed the analysis of the data and the writing of the manuscript. The search and inclusion of the literature were done by both authors.

## CONFLICTS OF INTEREST

The authors have no conflicts of interest to declare relevant to this article's content.

## FUNDING INFORMATION

This work was founded by the key medical and health projects in Xiamen (Grant Number: 3502Z20191104).

### PEER REVIEW

The peer review history for this article is available at https://publons.com/publon/10.1002/brb3.2850.

## Supporting information

Figure S1 Forest plots and SROC curves for AD versus NC subgroup

Figure S2 Forest plots and SROC curves for AD versus MCI subgroup

Figure S3 Forest plots and SROC curves for cMCI versus sMCI subgroup

Figure S4 Forest plots and SROC curves for ^11^C‐PIB PET subgroup

Figure S5 Forest plots and SROC curves for ^18^F‐AV45 PET subgroup

Figure S6 Forest plots and SROC curves for visual analysis subgroup

Figure S7 Forest plots and SROC curves for quantitative analysis subgroup

## Data Availability

All data are presented in this article. All data analysis and figures plotting were performed using R language, and the detailed code and data for analysis can be provided by contacting the corresponding author.
