# Supplementary figures and images for: Amyloid‐β PET in Alzheimer's disease: A systematic review and Bayesian meta‐analysis
Source: Brain Behav. 2022 Dec 27;13(1):e2850. doi: 10.1002/brb3.2850 (PMC9847612; doi:10.1002/brb3.2850)

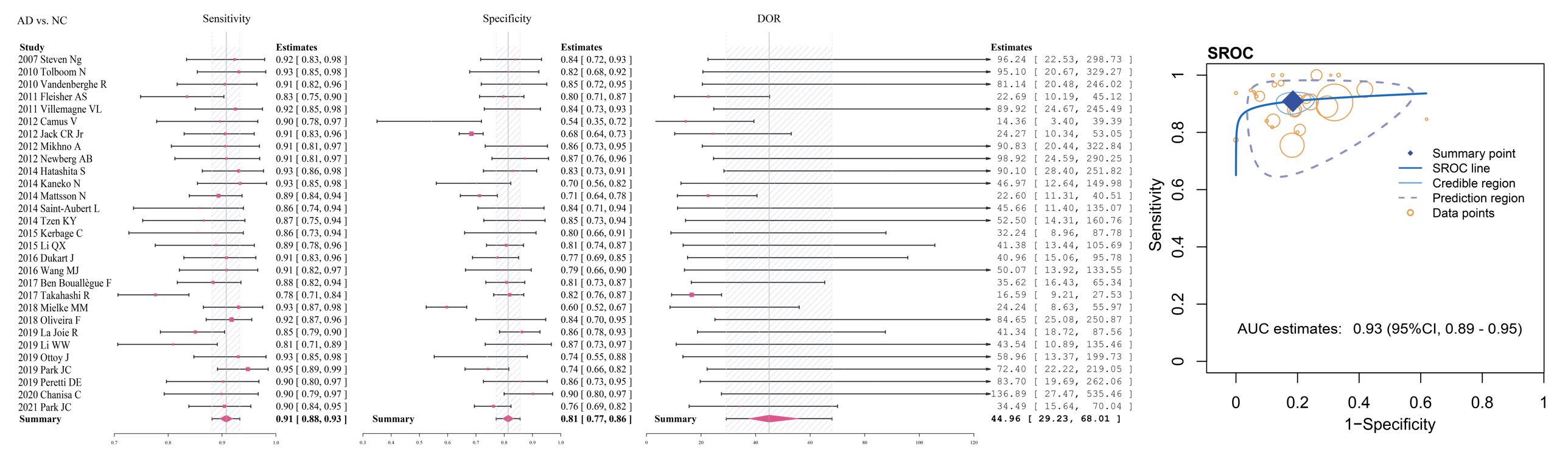

Supplement: Supplementary file 1 — Figure S1 Forest plots and SROC curves for AD versus NC subgroup [file BRB3-13-e2850-s004.tif]

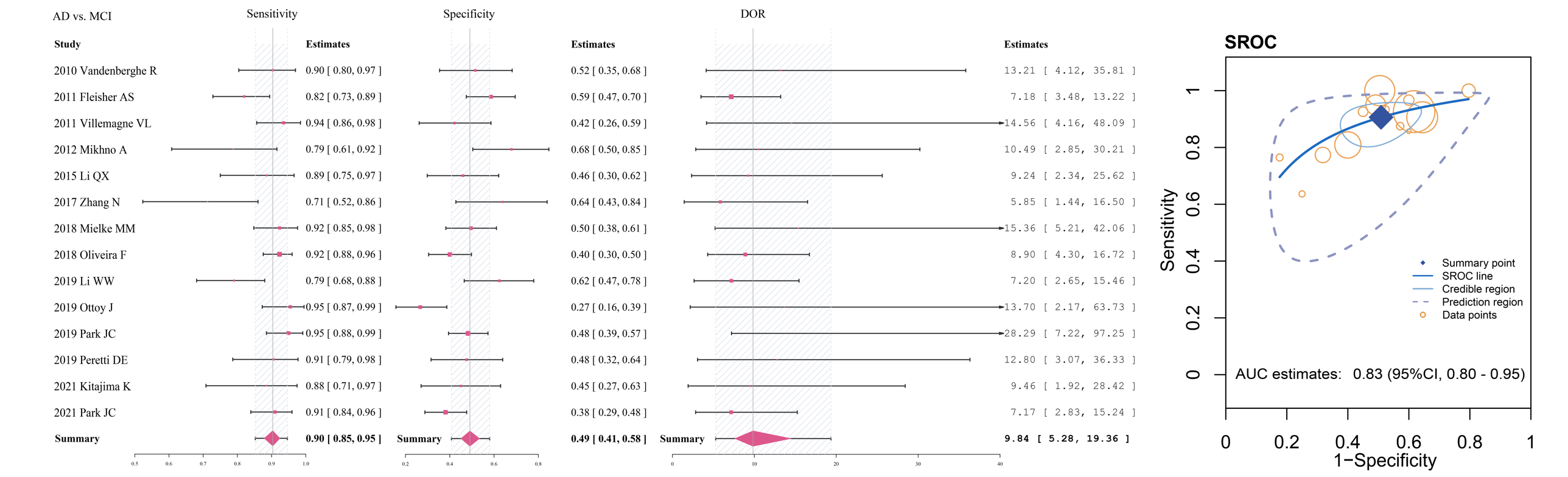

Supplement: Supplementary file 2 — Figure S2 Forest plots and SROC curves for AD versus MCI subgroup [file BRB3-13-e2850-s001.tif]

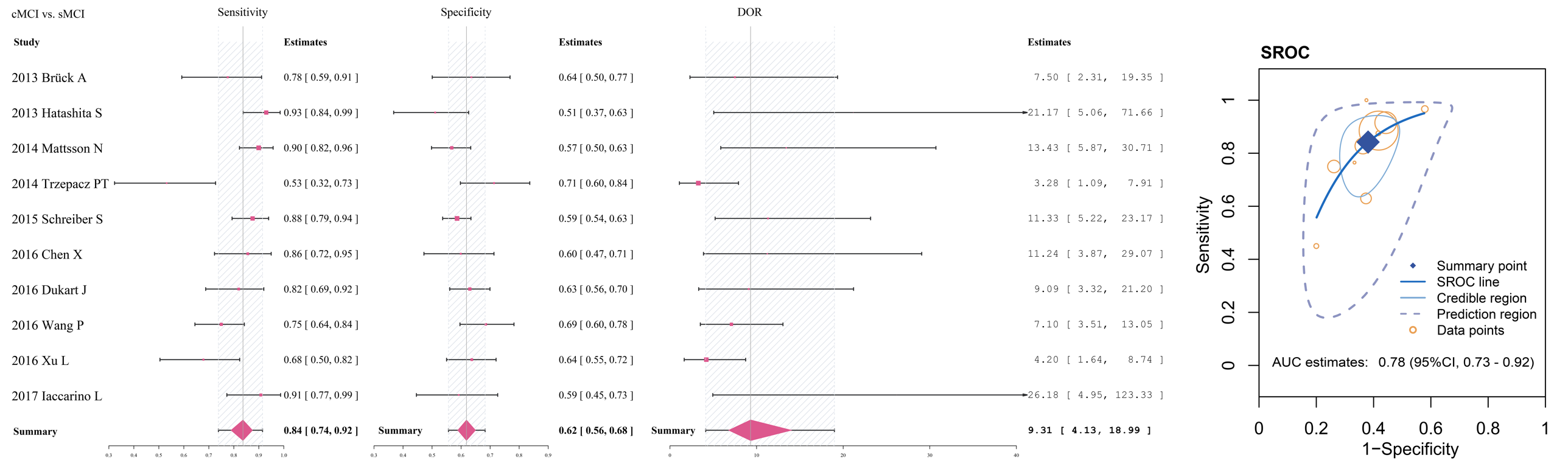

Supplement: Supplementary file 3 — Figure S3 Forest plots and SROC curves for cMCI versus sMCI subgroup [file BRB3-13-e2850-s002.tif]

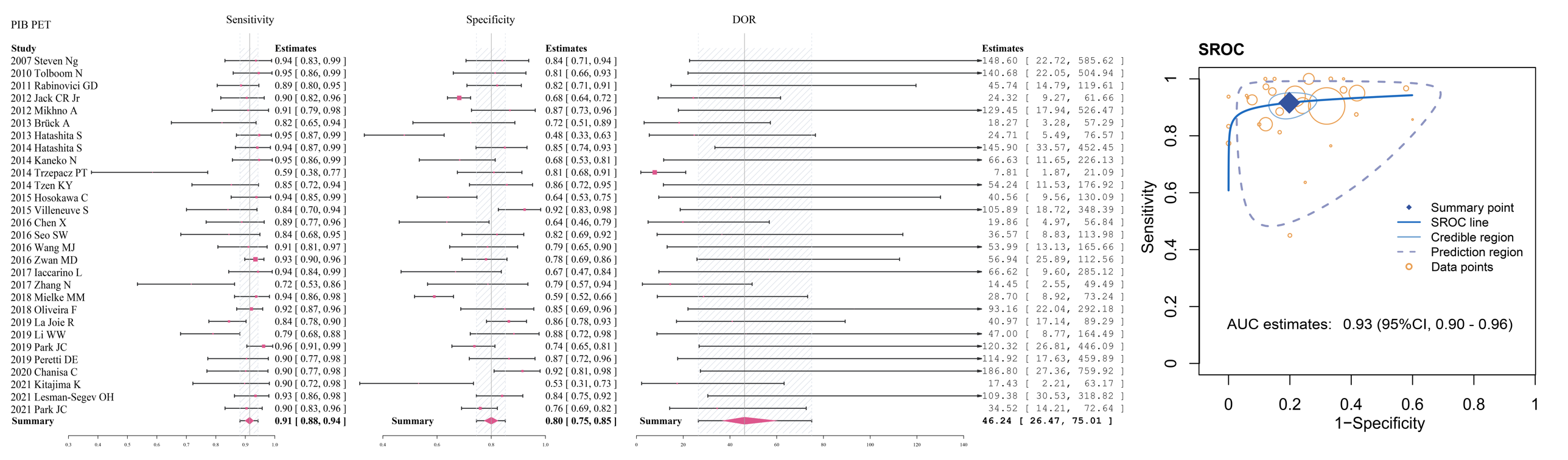

Supplement: Supplementary file 4 — Figure S4 Forest plots and SROC curves for 11C‐PIB PET subgroup [file BRB3-13-e2850-s003.tif]

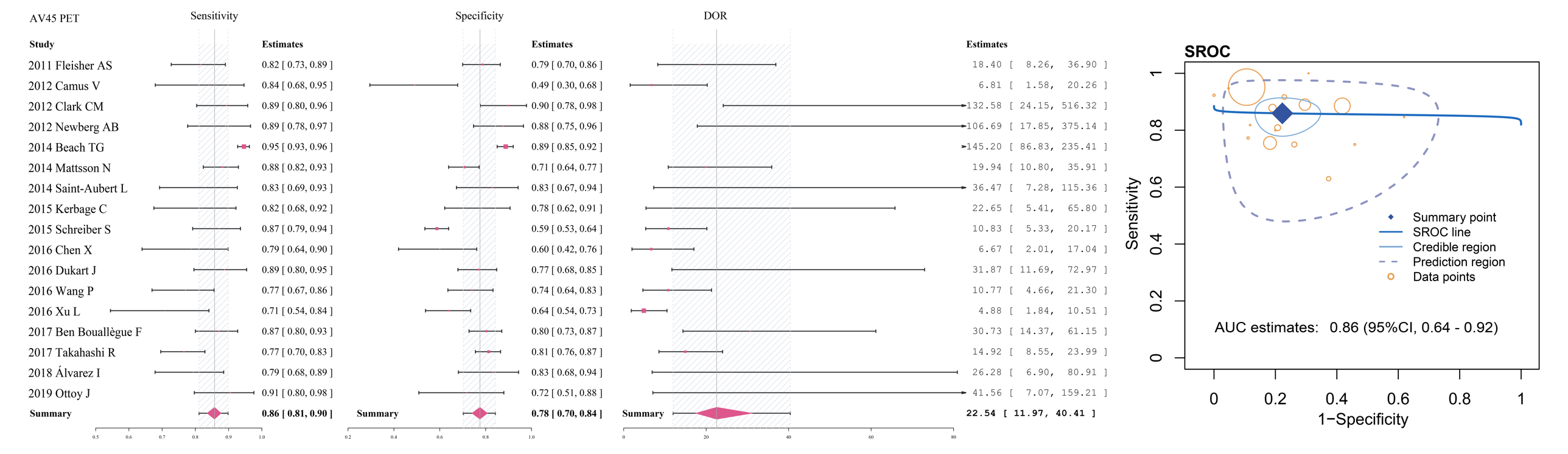

Supplement: Supplementary file 5 — Figure S5 Forest plots and SROC curves for 18F‐AV45 PET subgroup [file BRB3-13-e2850-s006.tif]

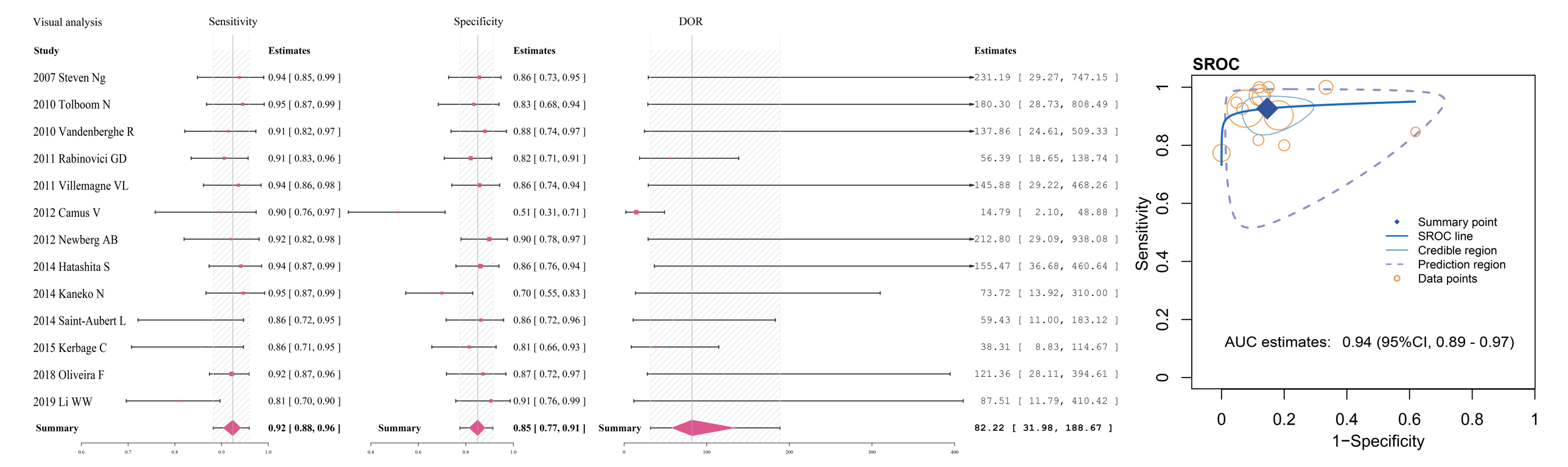

Supplement: Supplementary file 6 — Figure S6 Forest plots and SROC curves for visual analysis subgroup [file BRB3-13-e2850-s005.tif]

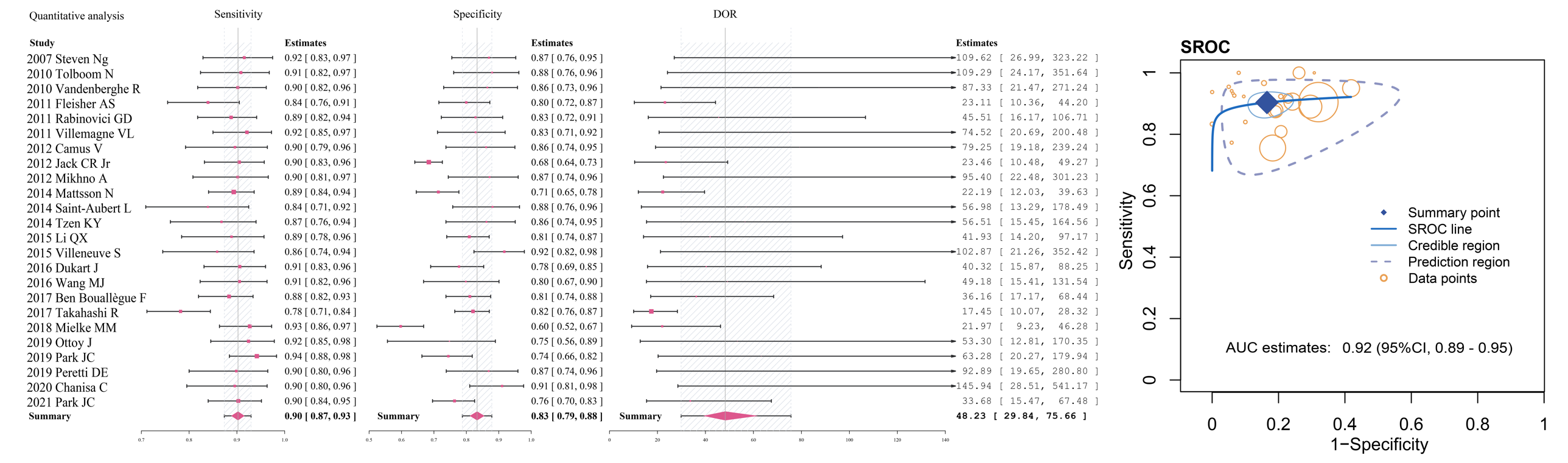

Supplement: Supplementary file 7 — Figure S7 Forest plots and SROC curves for quantitative analysis subgroup [file BRB3-13-e2850-s007.tif]
